# Appendiceal Neoplasm in Conservatively Managed Acute Appendicitis: A Retrospective Observational Study

**DOI:** 10.7759/cureus.70480

**Published:** 2024-09-30

**Authors:** Rahel Rashid, Baidar Khalabazyane, Israa Kadhmawi, Kamalesh Inteti, Matthew Woodhouse, Joseph Hanna

**Affiliations:** 1 General and Colorectal Surgery, Arrowe Park Hospital, Wirral, GBR; 2 Urology, Royal Bournemouth Hospital, Bournemouth, GBR; 3 Clinical Research fellow, Arrowe Park Hospital, Wirral, GBR; 4 General Surgery, Arrowe Park Hospital, Wirral, GBR; 5 Anaesthesia, Wirral University Teaching Hospitals, Liverpool, GBR; 6 Trauma and Orthopaedics, Wirral University Hospital, Cheshire, GBR

**Keywords:** appendiceal malignancy, appendiceal mass, appendiceal mucinous neoplasm, conservative management of acute appendicitis, non-operative management of acute appendicitis

## Abstract

Introduction

Appendiceal neoplasms are more prevalent in patients ≥ 40 years old who present with complicated appendicitis, especially if managed conservatively. Routine interval appendicectomy is not recommended. Follow-up bowel screening using both a CT scan and colonoscopy is recommended. Following the COVID-19 pandemic, many units have increased their utilization of non-operative management of acute appendicitis (NOM). This provides an optimal population sample to study the incidence of unexpected appendiceal malignancy compared to a similar cohort that underwent operative management. The primary outcomes of interest include the incidence of appendiceal malignancy following NOM, efficacy of bowel screening, and rates of re-admission.

Methods

A retrospective, observational study on patients admitted with acute appendicitis from January 2020 to December 2021. All patients diagnosed with acute appendicitis aged 40 years and older were included in the study, while those under 40 or without a diagnosis of acute appendicitis were excluded.

Results

We had 211 cases of acute appendicitis. 125 (59%) of which were managed operatively, while 86 cases (41%) were managed NOM. We found six cases (7%) of appendiceal malignancy in the NOM cohort, compared to two cases (1.6%) in the operatively-managed cohort. A routine follow-up colonoscopy failed to reveal any sinister pathology. All six cases underwent interval appendicectomy through which the malignancy was detected. 39 cases (45%) of NOM had at least one episode of re-admission, with 32 (37%) of them being in the first year.

Conclusion

NOM of acute appendicitis in adults ≥ 40 years old is associated with an increase in unexpected appendiceal malignancy, none of which were detected on follow-up colonoscopy. We emphasize the need for closer surveillance and potentially more aggressive follow-up strategies, including routine interval appendicectomy, for older patients undergoing NOM of acute appendicitis.

## Introduction

Appendicitis is one of the most common general surgical emergencies in the world. It is usually classified into simple/uncomplicated and complicated appendicitis (abscess, phlegmon, perforation). Appendicectomy is offered for uncomplicated appendicitis, with around 40,000 appendicectomies performed in the UK in 2023 [1].

Conservative management (or non-operative management (NOM)) is usually reserved for complicated appendicitis [2]. Non-operative or conservative management involves an initial course of intravenous (IV) antibiotics, then a transition to oral antibiotics, and interventional radiology (IR) drainage may be employed to manage collections [3]. The World Society of Emergency (WSES) recommends laparoscopic appendicectomy for complicated appendicitis [4], but this is still considered controversial because early appendicectomy for appendiceal abscess can be challenging technically and due to the presence of distorted anatomy, adhesive bowel loops, and the inflamed tissues can result in extensive resections [5,6]. It is, therefore, not surprising that most surgeons are more comfortable in a non-operative approach at first, and potential interval appendicectomy later when the inflammation has settled.

The necessity of interval appendicectomy following successful NOM of acute appendicitis has been a topic of considerable debate. WSES recommends against routine interval appendicectomy for patients <40 years old, citing the relatively low recurrence rate of 12%-24%. They argue that this recurrence rate does not justify the increased morbidity and costs associated with surgical intervention. However, appendiceal neoplasms are shown to be significantly more prevalent in patients who have undergone interval appendicectomy, with incidence rates ranging from 10% to 29%, particularly in patients over 40 years old. This has led some clinicians to advocate for routine interval appendicectomy in older patients, especially given the potential for malignancy, which might otherwise be missed if conservative management is continued without follow-up surgery [2,7,8].

To offset this inherent risk, WSES recommends colonic screening using full-dose CT scan and colonoscopy for patients >40 years old following successful NOM.

The aim of this study was to evaluate the efficacy of NOM, the incidence of appendiceal malignancy following NOM of acute appendicitis, and the efficacy of colonic screening in all patients >40 years old during a 2-year period at our institution.

## Materials and methods

This was a retrospective study of all patients 40 years or older who were diagnosed with acute appendicitis (using CT or ultrasound scan) during a 2-year period (January 2020 to December 2021) at a District General Hospital. Patient demographics were collected, including age and sex, type of management (operative vs non-operative), recurrence rate of appendicitis, rate of interval appendicectomy, histopathology data, and length of hospitalization. Statistical analysis was performed using Jamovi statistical software (version 2.5). Student t-test was used for quantitative data and a P-value of < 0.05 was considered statistically significant. Exclusion criteria: patients below 40 years old, and patients who did not have radiologic evidence of acute appendicitis (CT or ultrasound scan).

In our study, 'failed NOM' refers to cases where patients initially treated with NOM required an appendicectomy during the same hospital admission due to a lack of improvement. These cases are categorized under (emergency) interval appendicectomies in our analysis.

We use the term “emergency interval appendicectomy” for cases where appendicectomy was performed due to either failed NOM or recurrent appendicitis after a successful NOM and discharge. This contrasts with “routine interval appendicectomy,” which is performed on an elective basis.

## Results

Four hundred sixty-one (461) patients were admitted with acute appendicitis during the two years of our study. After applying the exclusion criteria: 241 patients were below 40 years old, and 9 patients did not have any radiologic evidence for acute appendicitis. The remaining 211 patients were included in our analysis. All 211 patients were admitted with a diagnosis of acute appendicitis using a CT scan. Mean age was 61 years (range: 41 to 93 years). One hundred ten (52%) were female. One hundred twenty-five (59%) underwent immediate appendicectomy (operative management), with a median time to operation of 18 hours from admission.

**Figure 1:**
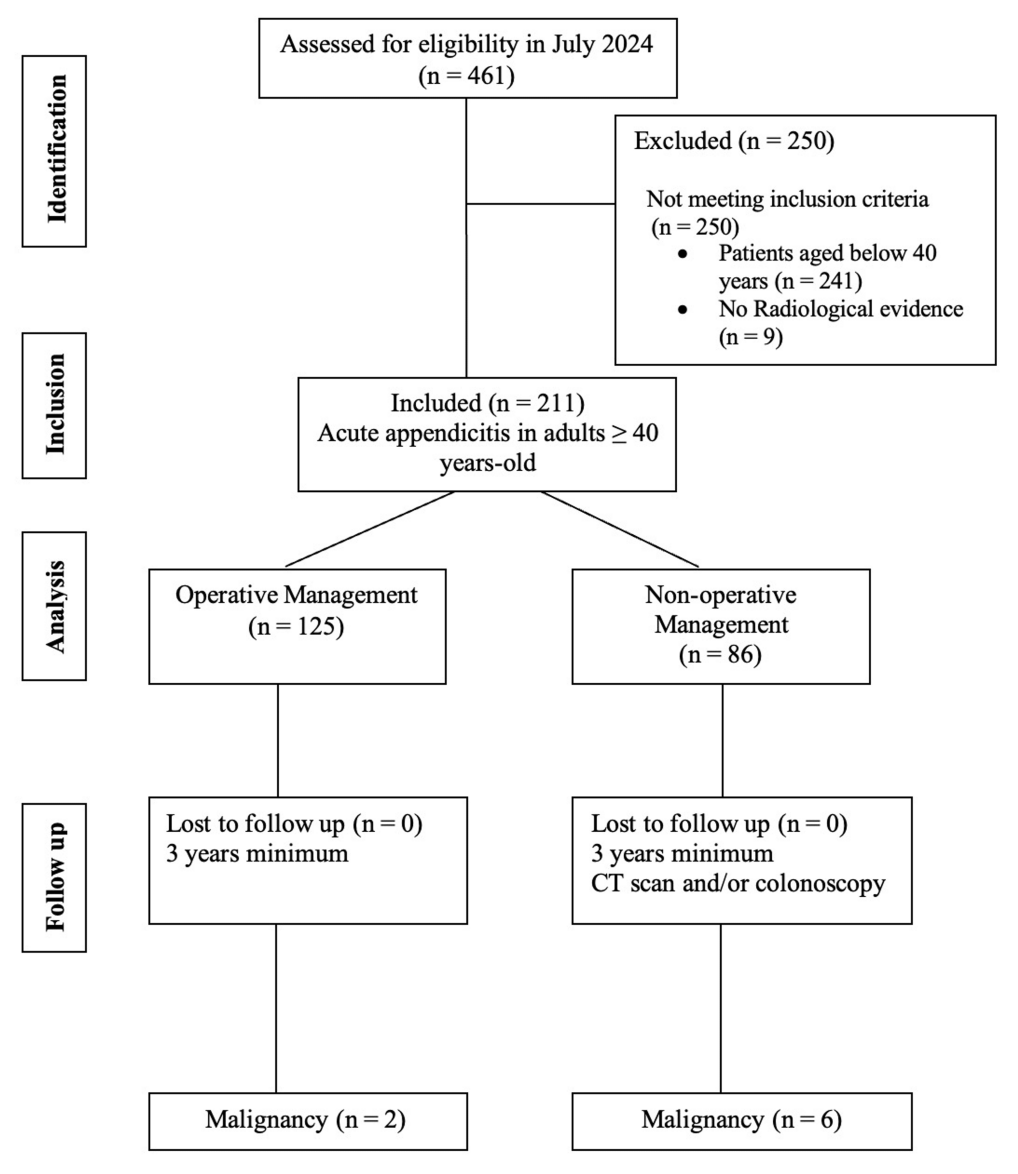
STROBE diagram showing the flow of patients n: number; CT: computed tomography; STROBE: Strengthening the Reporting of Observational Studies in Epidemiology

A Strengthening the Reporting of Observational Studies in Epidemiology (STROBE) diagram is shown below to document the flow of the patients (Figure 1).

Eighty-six cases (41%) from the cohort were offered NOM. From this, 48 (56%) patients were not offered and have not undergone interval appendicectomy; while 38 patients (44%) underwent interval appendicectomy, 12 (31.6%) of these were intended routine interval appendicectomy (i.e., on an elective list), and 26 (68.4%) were emergency interval appendicectomy. Most common cause for emergent interval appendicectomy was recurrent appendicitis (21 cases), followed by failure in NOM (5 cases). The mean time to interval appendicectomy was 32 weeks (range 1 week to 3 years).

Regarding recurrent appendicitis and re-admission: we had 39 cases (45%) of recurrent appendicitis in the NOM cohort. Of these, 32 (37%) cases were in less than one year following discharge from the hospital, and a further 7 (8%) within three years following discharge.

Nineteen cases (out of 38) were uncomplicated appendicitis; a further 19 cases were complicated appendicitis (nine cases of appendiceal abscess, three cases of gangrenous appendicitis, and seven cases of perforated appendicitis). Table 1 below summarizes the results.

**Table 1:** Cases that underwent interval appendicectomy CT: computed tomography; N: number

Diagnosis At Admission	N (%)	N Neoplasm Found
Uncomplicated appendicitis	19 (50)	4
Complicated appendicitis	19 (50)	2
Appendiceal abscess	9 (24)	2
Perforated appendicitis	7 (18)	0
Gangrenous appendicitis	3 (8)	0

The mean average length of hospital stay (LOS) for the operative cohort was 4.77 days, and 4.89 days for the NOM cohort. We found that 94% of the cases within the NOM cohort were successfully treated with a combination of IV and oral antibiotics. The most common antibiotic formulation used was IV cefuroxime and metronidazole. Interventional radiology (IR)-guided drainage of the peri-appendiceal collection was utilized in 14 cases.

We found two cases of appendiceal neoplasm from the operative group (125 cases - 1.6%). One case of appendiceal adenocarcinoma and a case of mucinous appendiceal neoplasm. On the other hand, we found six cases (6.9%) of malignancy in the NOM cohort (p-value 0.041). Only one case of neuroendocrine tumor, one of appendiceal adenocarcinoma, and three cases of mucinous appendiceal neoplasm. A histopathology report was not available for one of the cases. The mean age of patients diagnosed with appendiceal neoplasm was 67.5 years (range 55 to 79 years old).

The colonic screening was performed in 43 cases (50%): 21 cases (49%) with both CT scan and colonoscopy, 13 cases (30%) with colonoscopy only, and nine cases (21%) with CT scan. All of the cases in which malignancy were detected were offered bowel screening, and none of them were detected via colonic screening. The findings are shown below in Table 2.

**Table 2:** Summary of findings *student’s t-test; d: days; N/A: not applicable (p-value not calculated)

Outcome(s)	Operative Management	Non-operative Management	P-value*
Total number admissions	125	86	N/A
Re-admissions - N (%)	10 (8%)	39 (45%)	< 0.001
1 year	10	32 (37%)	
3 years	0	7 (8%)	
Length of stay (d)	4.77	4.89	0.092
Colonic screening	2	43	N/A
Neoplasm	2	6	0.045

## Discussion

The NOM of acute appendicitis has recently received renewed interest, especially during the COVID-19 pandemic, seen as a significant alternative to surgical management in uncomplicated acute appendicitis, with the WSES recommending it [4], and the Comparing Outcomes of Antibiotic Drugs and Appendectomy (CODA) trial showing its non-inferiority [9]. While appendicitis is a common symptom associated with appendiceal neoplasm, these neoplasms are a relatively rare cause of appendicitis. The prevalence of appendiceal neoplasms in patients undergoing NOM has been extensively studied and can serve as a compelling counterargument to NOM. This concern is particularly relevant when considering the recommendation against routine interval appendicectomy, as the risk of undetected neoplasms may outweigh the benefits of avoiding surgery. Higher rates of appendiceal neoplasm in cases that undergo interval appendicectomy have been well documented in the literature, such as the systematic review by Peltrini et al., which reported a rate of 11% [10].

Our study demonstrated a 7% incidence of appendiceal neoplasm in the NOM acute appendicitis cases, compared to a 1.6% incidence in operatively managed patients (p-value 0.41). The most prevalent subtype was mucinous appendiceal neoplasm, which is similar to the findings by Furman et al. [2]. Our study was a mixed cohort of conservatively managed complicated and uncomplicated acute appendicitis, and, surprisingly, we found a higher incidence of malignancy among the uncomplicated cohort, contrary to much of the literature [2,8-10]. This may be explained by the relatively long time (mean 32 weeks) after which interval appendicectomies were carried out at our institution.

The necessity of interval appendicectomy after successful NOM of acute appendicitis remains a topic of ongoing debate, especially since the reported recurrent appendicitis rate is relatively low (12-24%) and operative costs are taken into consideration [11,12]. A systematic review conducted by Darwazeh et al. revealed that interval appendicectomy and repeated NOM for cases of recurrent appendiceal phlegmon demonstrated similar rates of morbidity. Nonetheless, elective interval appendicectomy was found to incur supplementary operative costs, despite only succeeding in preventing recurrence in approximately one out of every eight patients. Based on these findings, the authors recommend against routine interval appendicectomy [13]. This is similar to another review by Anderson and Petzold, in which they reported an incidence rate of 7.4% of recurrent appendicitis [11]. These are in contrast to our finding of a 45% rate of recurrent appendicitis within three years of discharge from follow-up.

We are concerned that much of the literature may be underestimating the prevalence of appendiceal neoplasms in their study populations. This could be due to factors such as inadequate follow-up periods, the inclusion of pediatric patients or young patients aged <40 years, and the lack of histopathology specimens resulting from recommendations against interval appendicectomy. Similar to other studies, we observed a high rate of appendiceal neoplasms in the conservatively managed cohort. Worryingly, none of these cases were detected through the standard recommended follow-up strategies of CT scan and colonoscopy. Consequently, we believe these findings should prompt a reassessment of follow-up strategies in the older population. Regularly performing interval appendicectomy in patients above 40 years of age may be a more effective follow-up mechanism to detect appendiceal neoplasms and ensure better outcomes.

We recognize that our study has limitations. It was a retrospective, single-center study with a relatively small sample size of 211 patients, including only 38 interval appendectomies. Sample selection of both complicated and uncomplicated acute appendicitis could also be another limitation, along with the fact that we did not adjust for risk factors. Further randomized controlled trials (RCTs) that adjust for risk factors could provide valuable insights.

## Conclusions

We found a significantly higher incidence of appendiceal neoplasms in patients ≥40 years who underwent NOM of acute appendicitis compared to operative management. Current follow-up protocols (colonoscopy and CT scan) failed to detect these malignancies. These findings suggest that current guidelines against routine interval appendicectomy for older patients may need reconsideration. We emphasize the need for closer surveillance and potentially more aggressive follow-up strategies, including routine interval appendicectomy, for older patients undergoing NOM of acute appendicitis. Larger, prospective studies are needed to confirm these findings and inform future guidelines.
